# Cold Shock Proteins Balance Biofilm-Associated Antibiotic Resistance and Oxidative Vulnerability in Mycobacteria

**DOI:** 10.3390/microorganisms13071597

**Published:** 2025-07-07

**Authors:** Jiachen Zheng, Linzhao He, Yizhang Wei, Jie Lu, Xiaolin Liu, Weihui Li

**Affiliations:** State Key Laboratory for Conservation and Utilization of Subtropical Agro-Bioresources, College of Life Science and Technology, Guangxi University, Nanning 530004, China

**Keywords:** cold shock protein, biofilms, oxidative stress, isoniazid resistance, transcriptional regulation

## Abstract

Cold Shock Proteins (Csps) are multifunctional regulators critical for bacterial stress adaptation. While Csps are known to regulate biofilm formation and low-temperature growth in some species, their roles in mycobacteria remain unclear. Here, we explored the functions of three Csps (CspA1, CspA2, and CspB) in *Mycobacterium smegmatis*. We found that CspA1 promotes biofilm formation and isoniazid (INH) resistance but negatively affects oxidative stress resistance. In contrast, CspB promotes biofilm formation, whereas CspA2 appears functionally redundant in this process. Notably, CspB and CspA2 do not contribute redundantly to oxidative stress resistance. Proteomic analysis revealed that CspA1 significantly modulates the expression of key metabolic and stress-response proteins, including WhiB3 and KatG. Our findings establish CspA1 as a key regulatory factor in mycobacteria, linking metabolic adaptation to biofilm-associated drug resistance and oxidative defense.

## 1. Introduction

*Mycobacterium tuberculosis* (Mtb), the causative agent of tuberculosis (TB), is a leading global health threat, responsible for 1.25 million deaths in 2023, surpassing even COVID-19 mortality rates [1]. This resurgence has reinstated TB as the deadliest infectious disease worldwide. Biofilms, a key virulence factor in mycobacteria, serve as a protective barrier that enhances bacterial persistence and drug resistance, making their study critical for developing novel TB control strategies [2,3]. Analyzing the regulatory mechanism of mycobacterial biofilm formation is beneficial for developing more effective prevention and control measures.

Cold Shock Proteins (Csps) are emerging as multifunctional regulators of biofilm formation across bacterial species. In *Listeria monocytogenes*, Csps positively regulate biofilm formation by modulating bacterial motility [4]. In *Acinetobacter baumannii*, Csps act as novel transcription factors, coordinating virulence and biofilm formation [5]. In addition, in *Samonella Typhimurium*, CspE indirectly influences biofilm formation by downregulating *cspA* [6,7]. Despite these advances, the role of Csps in mycobacterial biofilms remains poorly understood, highlighting a critical knowledge gap.

Beyond biofilm regulation, Csps contribute to antibiotic resistance by modulating membrane and efflux systems. In *Escherichia coli*, CspA upregulates the expression of AcrAB-TolC efflux pumps, enhancing multidrug resistance [8]. Similarly, in *Staphylococcus aureus*, *cspA* expression levels correlate with vancomycin susceptibility, implicating it in cell wall maintenance and stress adaptation [9,10]. However, whether Csps play a comparable role in mycobacteria remains unexplored.

Oxidative stress, a major constraint on Mtb growth in vivo, is a key host defense mechanism [11,12]. In *E. coli*, CspA stabilizes mRNA transcripts of antioxidant enzymes (e.g., superoxide dismutase (SOD) and catalase), enhancing resistance to reactive oxygen species (ROS) [13]. In *Staphylococcus aureus*, *cspA* depletion disrupts metabolic and stress-response pathways, impairing oxidative stress tolerance [14]. Despite these findings, the mechanisms by which Csps mediate oxidative stress adaptation in mycobacteria are unknown.

Here, we investigated the functional diversity of three mycobacterial Csps (CspA1, CspA2, and CpsB) in *Mycobacterium smegmatis*. We demonstrate that CspA1 and CspB promote biofilm formation, whereas CspA2 is dispensable in this process. Strikingly, Csps collectively suppress oxidative stress tolerance, with CspA1 additionally enhancing INH resistance. Proteomic analysis reveals that CspA1 modulates key stress-response proteins, suggesting a transcriptional regulatory role. These findings revealed the specialized functions of mycobacterial Csps, providing new insights into their role in stress adaptation and drug resistance.

## 2. Materials and Methods

### 2.1. Strains and Growth Conditions

*M. smegmatis* (Msm) strains were cultured in 7H9 liquid medium (BD Difco, Franklin Lakes, NJ, USA) supplemented with 4.7 g/L Middlebrook 7H9 (2.5 mL/L 20% Tween80 and 4 mL/L 50% glycerol) or on 7H10 solid medium supplemented with 1.9 g/100 mL (Middlebrook 7H10 (BD Difco, Franklin Lakes, NJ, USA) and 1 mL/100 mL 50% glycerol). *E. coli* strains were grown in LB liquid medium (10 g/L tryptone (OXOID, Hampshire, UK), 10 g/L sodium chloride (Sangon Biotech, Shanghai, China), and 5 g/L yeast powder (OXOID, Hampshire, UK) with 1.5 g/100 mL agar (Sangon Biotech, Shanghai, China)) for solid plates.

### 2.2. Generation of Mutants, Complementation, and Constitutive Expression Plasmids

The knockout mutants (Δ*cspA1*, Δ*cspA2*, Δ*cspB*) were generated in *M. smegmatis* mc^2^155 using homologous recombination. This involved constructing a suicide plasmid (pMind) carrying a hygromycin resistance gene (*hygR*) and a *lacZ* gene for blue-white screening with X-gal. Upstream and downstream homologous flanking regions of each target *csp* gene were amplified (primers in Table S1), digested alongside the pMind vector with specific restriction enzymes, and ligated to create the knockout construct. This construct was electroporated into wild-type *M. smegmatis*, and transformants were selected on hygromycin. Double-crossover events, confirmed by white colony formation on X-gal plates, replaced the genomic csp sequence with the deletion construct.

For the complementation strains Δ*cspA1*-comp, Δ*cspA2*-comp, and Δ*cspB*-comp, the respective wild-type *csp* gene was cloned into the vector pMV261 (conferring kanamycin resistance, kanR), generating plasmids pMV261-*cspA1*, pMV261-*cspA2*, and pMV261-*cspB*. Each plasmid was then electroporated into its corresponding isogenic knockout strain, and transformants were selected on kanamycin. Constitutive overexpression strains (WT::*cspA1*, WT::*cspA2*, WT::*cspB*) were similarly generated by electroporating these pMV261-*csp* plasmids into the wild-type *M. smegmatis* strain, followed by kanamycin selection.

### 2.3. Colony Morphology Experiments

Bacterial cultures (OD_600_ = 1.0 in 7H9 medium) were spotted or streaked onto 7H10 agar plates and incubated at 37 °C for three days. Colonies were examined for size, surface texture (smooth, rough, mucoid, wrinkled, and morphology). Colony Morphology were documented using standardized macroimaging.

### 2.4. Static Biofilm Phenotyping Experiments

For the air–liquid surface biofilm growth assay, strains were grown in 7H9 medium to OD_600_ = 1.0 and then adjusted to OD_600_ = 0.3 in M63 medium (100 mM KH_2_SO_4_ (Solarbio, Beijing, China), 75 mM KOH (Tianjin Beichen Fangzheng Reagent Factory, Tianjin, China), 15 mM (NH_4_)_2_SO_4_ (Sinopharm Chemical Reagent Co., Ltd., Shanghai, China), 2 mM MgSO_4_ (Solarbio, Beijing, China), 3.9 μM FeSO_4_ (Solarbio, Beijing, China), 2% glucose (*m*:*V*) (Tianjin AoboKai Chemical Co., Ltd., Tianjin, China), 0.5% casein hydrolysate (*m*:*V*) (Macklin, Shanghai, China), 0.7 Mm CaCl_2_ (Tianjin Aopusheng Chemical Co., Ltd., Tianjin, China), transferred to 12-well plates (2.5 mL/well), and incubated at 30 °C.

### 2.5. Crystal Violet Staining Quantitative Assay

Strains were grown to OD_600_ = 1.0 in 7H9 medium. Cell cultures were then adjusted to OD_600_ = 0.1 in M63 medium and added to 96-well plates (100 μL/well), incubating at 37 °C for 80 rpm for 36 h. The supernatants were removed and left to dry. Then, 120 μL/well 0.1% (*m*:*V*) crystal violet (Solarbio, Beijing, China) was used to stain the biofilms for 30 min. After washing twice with distilled water (200 μL/well) and air-drying, 200 μL/well ethanol/acetone mixture (80% ethanol + 20% acetone, *V*:*V*) was added to dissolve crystal violet for 5 min at room temperature. The absorbance value was detected at the 570 nm wavelength.

### 2.6. INH Susceptibility Testing Experiments

*Mycobacterium smegmatis* strains were grown overnight in Middlebrook 7H9 broth supplemented with 0.2% glycerol and 0.05% Tween 80. Kanamycin (30 µg/mL) (Solarbio, Beijing, China) was included for plasmid-bearing strains (complementation and constitutive overexpression strains) to maintain plasmid integrity. Cultures in the mid-exponential phase (OD_600_ = 0.8–1.2) were diluted in fresh 7H9 broth to an initial OD_600_ of 0.1. Aliquots (100 mL) of this standardized inoculum were dispensed into flasks containing specific concentrations of isoniazid (INH) or other tested antibiotics (concentrations detailed in Figure Legends/Results). Cultures were incubated at 37 °C with shaking (160 rpm). At different time points, serial dilutions of samples were plated on 7H10 plates for counting colony-forming units (cfus). Aliquots were taken at the indicated times. Biological triplicates were performed for each strain and condition. Growth curves were plotted, and statistical analysis (e.g., Student’s *t*-test, ANOVA) was applied where appropriate to compare strain sensitivities.

### 2.7. Oxidative Stress Experiments

For the oxidative stress experiments, cultures were divided into two groups, experimental versus control groups, to determine mycobacterial growth curves and the effect of hydrogen peroxide; *M. smegmatis* was grown overnight in Middlebrook 7H9 media (complemented with 0.05% Tween-80 and 0.2% glycerol) containing 30 μg/mL Kan. When cells entered a growth phase (OD_600_ between 0.8 and 1.2), the OD of each culture was adjusted to 0.1 in 100 mL of fresh 7H9 broth containing the indicated concentration of hydrogen peroxide. The cultures were then allowed to grow further at 37 °C with shaking at 160 rpm. At different time points, serial dilutions of samples were plated on 7H10 plates for counting colony-forming units (cfus). Aliquots were taken at the indicated times.

### 2.8. Proteomic Assays

Biological triplicates of wild-type *M. smegmatis* mc^2^155 and the Δ*cspA1* mutant were cultured in Middlebrook 7H9 medium supplemented with 0.05% Tween 80 to the mid-exponential phase (OD_600_ = 1.0) at 37 °C with shaking (160 rpm). To ensure precisely matched metabolic states, cultures were then diluted to OD_600_ = 0.1 in fresh medium and re-incubated until re-reaching OD_600_ = 1.0. Cells were immediately chilled on ice, harvested by centrifugation (4 °C, 6000× *g*, 10 min), and pellets were weight-balanced across strains before washing 2–3 times with ice-cold phosphate-buffered saline (PBS, pH 7.4) to remove residual medium components. After taking the appropriate amount of samples for grinding, the protein was extracted, and its concentration was determined. Following overnight enzymatic digestion in equal amounts, the protein samples were reduced with dithiothreitol (DTT) at 56 °C for 30 min. Subsequently, iodoacetamide (IAA) was added (11 mM final concentration), and the mixture was incubated at room temperature for 15 min in the dark. The peptides were then dissolved in liquid chromatography mobile phase and separated via the ultrahigh-performance liquid chromatography (UHPLC) system. Following UHPLC separation, peptides were ionized via nanoelectrospray ionization (NSI) and analyzed by Orbitrap Exploris 480 mass spectrometry. The NSI voltage was 2.3 kV, with FAIMS compensation voltages set to −45 V and −65 V. A high-resolution Orbitrap analyzed both parent ions and secondary peptide fragments, with the primary MS scan range set to 400–1200 *m*/*z* at 60,000 resolution. Proteomic profiling was executed by Hangzhou Jingjie Biotechnology Corporation, Hangzhou, China.

### 2.9. Statistics

Statistical analysis was performed using GraphPad Prism 7 (GraphPad Software Inc., San Diego, CA, USA). All the data shown are mean ± standard deviation (SD) from at least three biological replicates. Statistical significance was determined by Student’s *t*-test. *p* < 0.05 was considered statistically significant.

## 3. Results

### 3.1. M. smegmatis Genome Has Three Genes Encoding Csp Proteins

**Figure 1:**
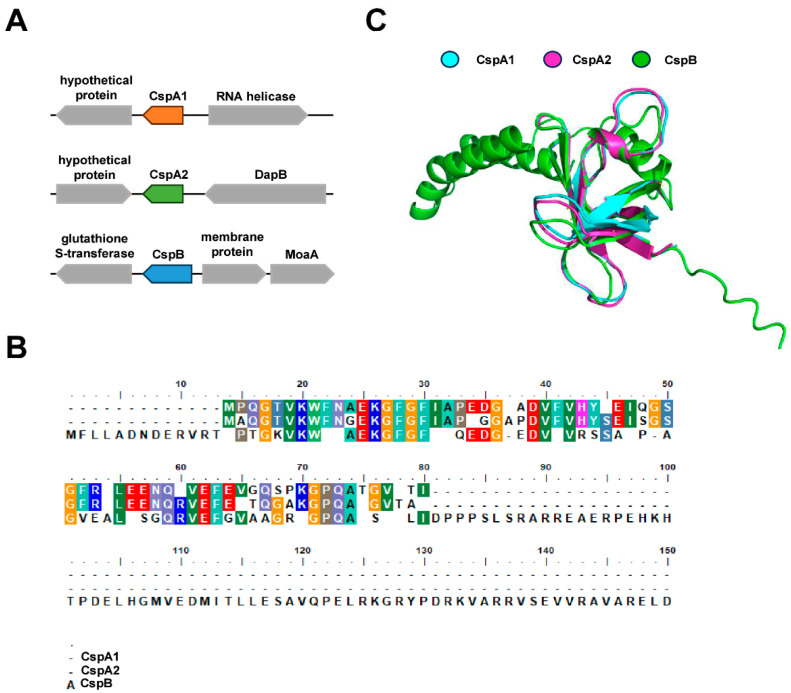
Structural characteristics of three Csp proteins. (**A**) Distribution characteristics of CspA1, CspA2, and CspB clusters. (**B**) Amino acids sequence alignment of Csp proteins via Bio Edit. The same color in each column represents the same amino acid, and one color represents the type of amino acid. (**C**) Structural alignment of three Csp proteins via using Alphafold 3.

Genome analysis of *M. smegmatis* identified three genes encoding cold shock proteins: *cspA1*, *cspA2*, and *cspB*. These genes exhibit distinct genomic organization, suggesting potential involvement in divergent metabolic pathways (Figure 1A). Sequence alignment revealed high amino acid conservation: CspA1 and CspA2 shared 84% identity, while pairwise comparisons of CspA1/CspB and CspA2/CspB both exhibited 65% identity (Figure 1B). Despite this sequence homology, AlphaFold-predicted structural models revealed marked divergence in secondary structure composition: CspB contains additional α-helices and random coils compared to CspA1 and CspA2 (Figure 1C). These structural variations, emerging even amid sequence conservation, suggest non-redundant biological roles for the three Csps.

### 3.2. CspA1 Affects Colony Morphology in Mycobacteria

**Figure 2:**
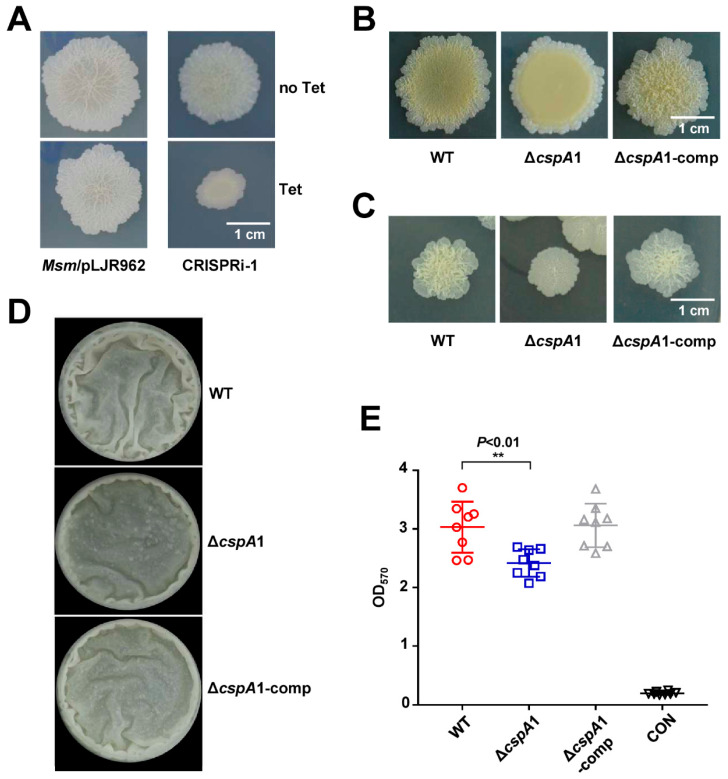
CspA1 affects colony morphology. (**A**) Colony phenotype of CRISPRi-*cspA1* strain. Top: CRISPRi-*cspA1* strain without tetracycline. Bottom: CRISPRi-*cspA1* strain with tetracycline. (**B**,**C**) Colony phenotype of wild-type, Δ*cspA1*, and Δ*cspA1*-comp strain on spot plates (**B**) and dilution plate coating (**C**). (**D**) Detection of biofilms on gas–liquid interface. Qualitative analysis of the wild-type, Δ*cspA1*, and Δ*cspA1*-comp strain using gas–liquid interface biofilm. (**E**) Quantitative of crystal violet. Measure the total amount of biofilm wild-type, Δ*cspA1,* and Δ*cspA1*-comp strain at an absorbance of 570 nm. Two-tailed Student’s *t*-tests were performed for statistical analysis of eight independent biological experiments (*p* < 0.01, **).

Our CRISPRi library screen identified *cspA1* as a candidate gene influencing mycobacterial colony morphology (Figure 2A). To validate this, we constructed a *cspA1* knockout strain (Δ*cspA1*) and its complementary strain (Δ*cspA1*-comp). The Δ*cspA1* exhibited a smoother colony architecture compared to the rough, wrinkled morphology of the wild-type colony, and the phenotype was fully rescued in the Δ*cspA1*-comp strain (Figure 2B,C). These results suggest that CspA1 may contribute to the rough morphology of mycobacterium.

### 3.3. CspA1 Promotes Biofilm Formation

Given the altered colony morphology of Δ*cspA1*, we assessed its biofilm-forming capacity. At the air–liquid interface, Δ*cspA1* displayed fewer biofilms compared to WT (Figure 2D). Crystal violet quantification confirmed a significant reduction in biofilm biomass in Δ*cspA1*, which was restored to WT-levels in the Δ*cspA1*-comp strain (Figure 2E). These results establish CspA1 as a key regulator of mycobacterial biofilm formation.

### 3.4. CspA1 Suppresses the Antioxidant Growth of Mycobacteria

**Figure 3:**
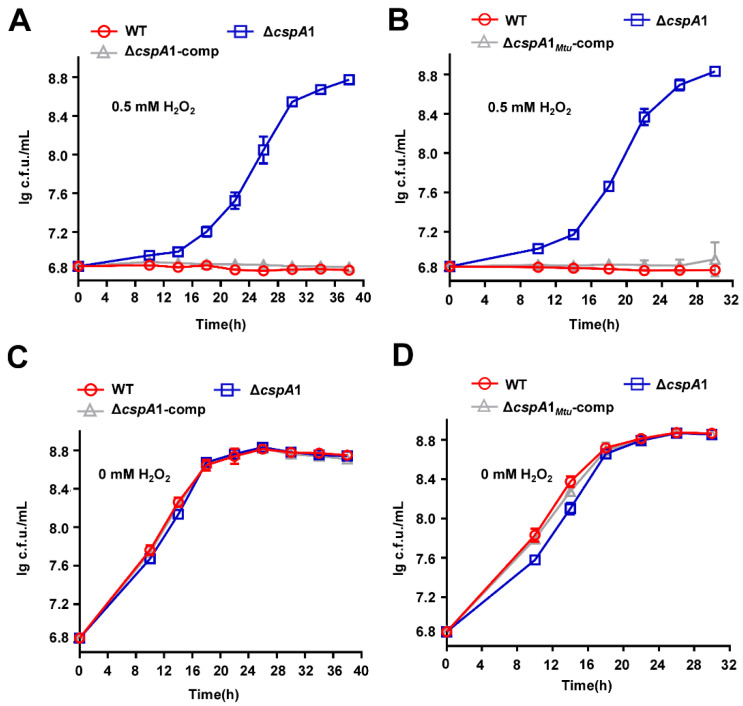
CspA1 regulates the antioxidant growth of mycobacteria. (**A**) Assays for the effect of oxidative stress on the growth of *M. smegmatis*. The mycobacterial strains, wild-type, Δ*cspA1*, and Δ*cspA1*-comp strain, were grown in 7H9 medium with 0.5 mM hydrogen peroxide. (**B**) Assays for the effect of oxidative stress on the wild-type, Δ*cspA1*, and Δ*cspA1*-comp strain were grown in 7H9 medium without hydrogen peroxide. (**C**) Assays for the effect of oxidative stress on the wild-type, Δ*cspA1*, and Δ*cspA1_Mtu_*-comp strain were grown in 7H9 medium with 0.5 mM hydrogen peroxide. (**D**) Assays for the effect of oxidative stress on the wild-type, Δ*cspA1*, and Δ*cspA1_Mtu_*-comp strain were grown in 7H9 medium without hydrogen peroxide.

Proteomic profiling revealed that CspA1 regulates a broad regulon, with 400 proteins upregulated and 402 downregulated in the Δ*cspA1* strain (Figure S1). This extensive rewiring suggests that CspA1 functions as a master RNA chaperone, coordinating transcriptional responses to balance stress adaptation programs. Notably, pathway enrichment analysis identified significantly differential expression in redox homeostasis pathways, including upregulated levels of five oxidoreductases and WhiB3, a novel iron–sulfur cluster protein, regulates redox homeostasis (Figure S2, Table S2), prompting us to investigate CspA1’s role in redox homeostasis. Under hydrogen peroxide stress, Δ*cspA1* exhibited significantly enhanced growth compared to wild-type, whereas the complemented strain Δ*cspA1*-comp was restored to wild-type level sensitivity (Figure 3A). No growth differences were observed among strains in the absence of hydrogen peroxide (Figure 3B), confirming that the phenotype is stress-specific.

Given the clinical relevance of oxidative defense in *M. tuberculosis*, we assessed the conservation of CspA1 function. Sequence alignment showed 92.5% amino acid identity between *M. smegmatis* CspA1 and its *M. tuberculosis* ortholog (CspA1_Mtu_) (Figure S3). Strikingly, heterologous overexpression of *cspA1*_Mtu_ in the Δ*cspA1* strain (Δ*cspA1*-*cspA*_Mtu_) fully rescued the oxidative stress-sensitive phenotype to WT levels (Figure 3C), with no fitness cost under non-stress conditions (Figure 3D). These results demonstrate that CspA1-mediated suppression of oxidative tolerance is evolutionarily conserved in mycobacteria.

### 3.5. CspA1 Contributes to the INH Resistance of Mycobacteria

**Figure 4:**
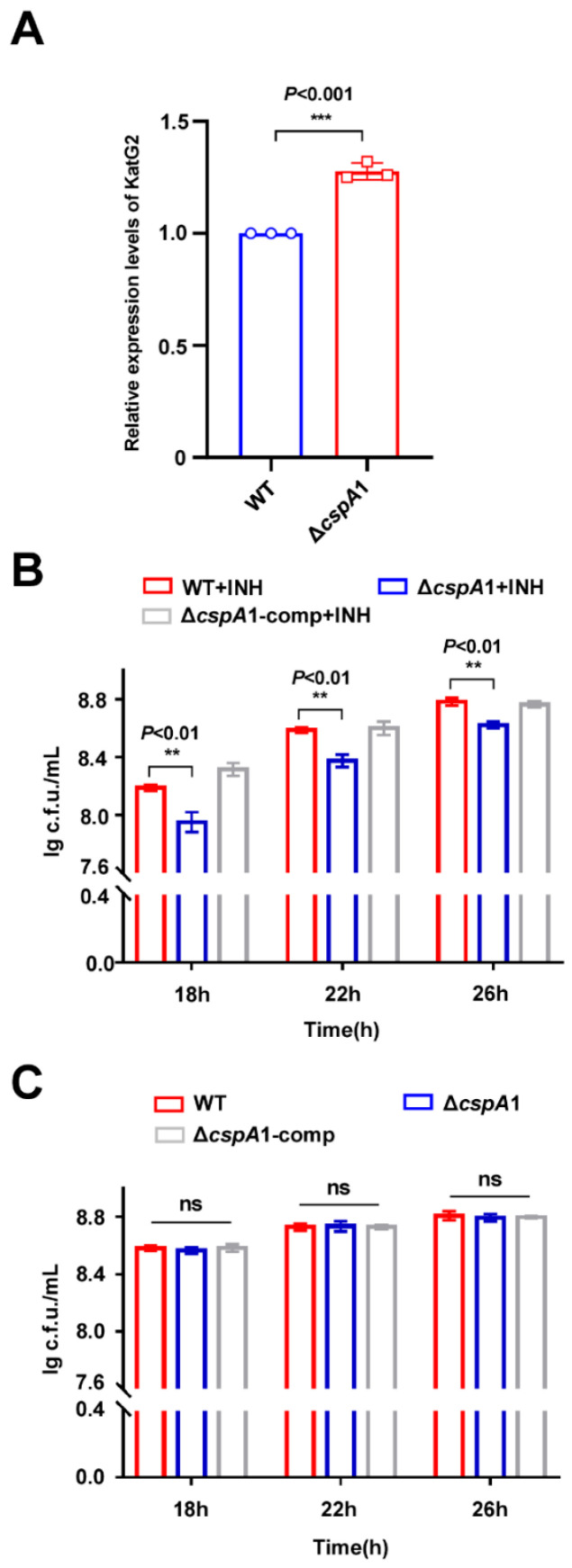
CspA1 positively regulates the INH resistance of mycobacteria. (**A**) Protein expression levels of KatG in wild-type and *cspA* knockout strains. (**B**) Assays for the effect of INH on the growth of *M. smegmatis*. The mycobacterial strains, wild-type, Δ*cspA1*, and Δ*cspA1*-comp strain were grown in 7H9 medium with INH. (**C**) Assays for the effect on the wild-type, Δ*cspA1*, and Δ*cspA1*-comp strain were grown in 7H9 medium without INH. Error bars represent the variant range of the data derived from three biological replicates. Two-tailed Student’s *t*-tests were performed for statistical analysis of three independent biological experiments (ns, not significant; *p* < 0.01, **; *p* < 0.001, ***).

Proteomic profiling of the Δ*cspA1* strain revealed a marked upregulation of KatG2, a catalase-peroxidase critical for INH activation in mycobacteria (Figure 4A). This suggests that CspA1 may regulate the INH resistance of mycobacteria. Δ*cspA1* exhibited increased INH susceptibility compared to wild-type, and complementation (Δ*cspA1*-comp) restored INH resistance to WT levels (Figure 4B). No growth defects were observed in drug-free medium (Figure 4C). Taken together, these findings indicate that CspA1 positively regulates mycobacterial INH resistance.

### 3.6. CspA2 Family Proteins Are Redundant in Biofilm Formation

**Figure 5:**
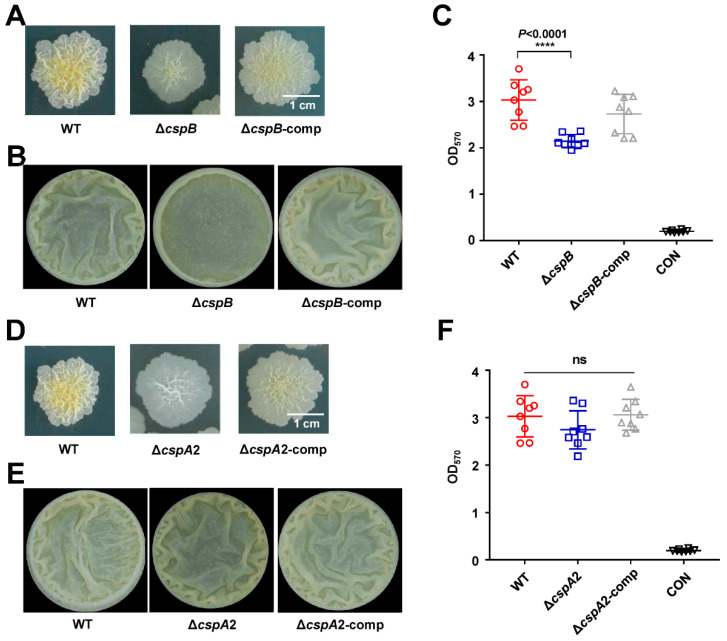
CspB and CspA2 affect colony morphology. (**A**) The impact of *cspB* knockout on surface morphology. Surface morphology of the mycobacterial strains, wild-type, Δ*CspB,* and Δ*cspB*-comp strain on 7H10. (**B**) Detection of biofilms on gas–liquid surfaces. Qualitative analysis of the wild-type, Δ*cspB,* and Δ*cspB*-comp strain using gas–liquid surface biofilm. (**C**) Quantitative of crystal violet. Measure the total amount of biofilm wild-type, Δ*cspB,* and Δ*cspB*-comp strain at an absorbance of 570 nm. (**D**) The impact of *cspB* knockout on surface morphology. Surface morphology of the mycobacterial strains, wild-type, Δ*cspA2,* and Δ*cspA2*-comp strain on 7H10. (**E**) Detection of biofilms on gas–liquid surfaces. Qualitative analysis of the wild-type, Δ*cspA2*, and Δ*cspA2*-comp strain using gas–liquid surface biofilm. (**F**) Quantitative of crystal violet. Measure the total amount of biofilm wild-type, Δ*cspA2*, and Δ*cspA2*-comp strain at an absorbance of 570 nm. Two-tailed Student’s *t*-tests were performed for statistical analysis of three independent biological experiments (*p* < 0.0001, ****; ns, not significant).

To assess functional redundancy among *M. smegmatis* Csps, we extended our analysis to CspB and CspA2. Building on the biofilm-defective phenotype of Δ*cspA1* (Figure 2), we generated *cspB* and *cspA2* knockout strains (Δ*cspB*, Δ*cspA2*) alongside complemented controls (Δ*cspB*-comp, Δ*cspA2*-comp). Δ*cspB* recapitulated the biofilm deficiencies observed in Δ*cspA1*: the Δ*cspB* exhibited smoother colony morphology (Figure 5A) and reduced air–liquid interface biofilm with fewer wrinkles (Figure 5B) compared to wild-type. Crystal violet assay confirmed the significant decrease in biofilm biomass of Δ*cspB*, which was fully restored in the Δ*cspB*-comp strain (Figure 5C).

In stark contrast, Δ*cspA2* exhibited WT-like biofilm morphology (Figure 5D), air–liquid biofilms with wrinkles (Figure 5E), and biofilm biomass (Figure 5F), despite sharing 83.58% sequence identity with CspA1. This functional divergence highlights specialized roles within the Csp family: while CspA1 and CspB are critical for biofilm formation, CspA2 appears to have evolved distinct regulatory targets.

### 3.7. CspA2 and CspB Negatively Regulate Anti-Oxidative Stress of Mycobacteria

**Figure 6:**
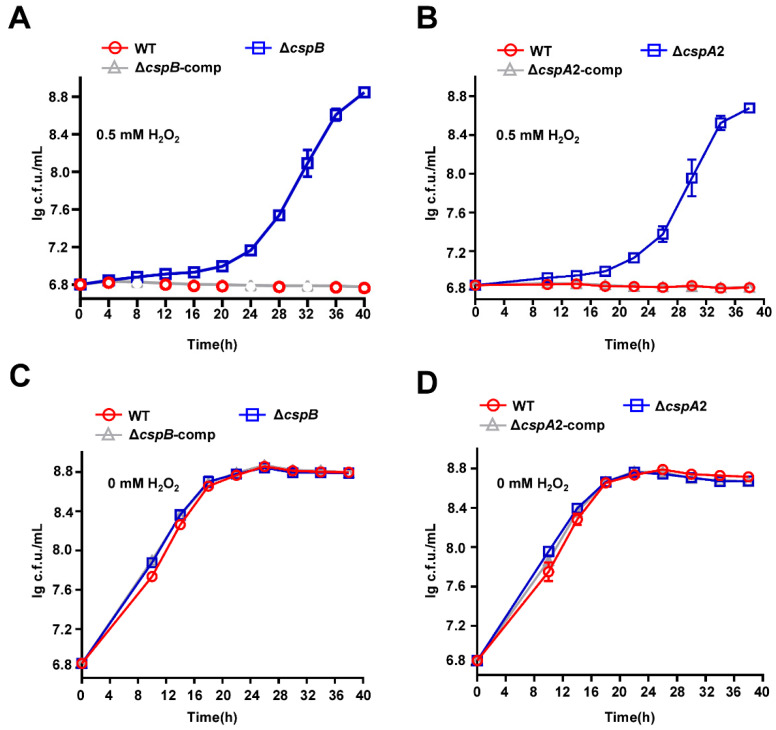
CspB and CspA2 regulate antioxidant growth of mycobacteria. (**A**) Assays for the effect of oxidative stress on the growth of *M. smegmatis*. The mycobacterial strains, wild-type, Δ*cspB*, and Δ*cspB*-comp strain, were grown in 7H9 medium with 0.5 mM hydrogen peroxide. (**B**) Assays for the effect of oxidative stress on the wild-type, Δ*cspA2*, and Δ*cspA2*-comp strain were grown in 7H9 medium with 0.5 mM hydrogen peroxide. (**C**) Assays for the effect of oxidative stress on the wild-type, Δ*cspB*, and Δ*cspB*-comp strain were grown in 7H9 medium without hydrogen peroxide. (**D**) Assays for the effect of oxidative stress on the wild-type, Δ*cspA2*, and Δ*cspA2*-comp strain were grown in 7H9 medium without hydrogen peroxide. Error bars represent the variant range of the data derived from three biological replicates.

To determine whether CspA2 and CspB share CspA1’s role in oxidative stress regulation (Figure 3), we analyzed Δ*cspB* and Δ*cspA2* under hydrogen peroxide challenge. Both mutants exhibited enhanced oxidative stress tolerance (Figure 6A,B), and complementation restored WT-like sensitivity in both strains (Figure 6A,B). No growth difference was observed among wild-type, mutant, and complemented strains without oxidative pressure (Figure 6C,D).

## 4. Discussion

Mycobacteria encounter multifaceted environmental challenges within host cells, including oxidative stress, antibiotic pressure, and nutrient deprivation. While cold shock proteins (Csps) are increasingly recognized as pivotal regulators of bacterial stress adaptation [15], their functional roles in mycobacteria remain poorly understood. Our systematic investigation of three Csps (CspA1, CspA2, and CspB) in *M. smegmatis* reveals associations between their absence and altered biofilm maturation, oxidative stress tolerance, and antibiotic resistance—three hallmark mechanisms of mycobacterial persistence.

Both CspA1 and CspB deletions correlated with impaired biofilm maturation, with Δ*cspA1* and Δ*cspB* mutants exhibiting pronounced defects in colony morphology and biomass accumulation (Figure 2 and Figure 5). While Csps might modulate biofilm formation through conserved metabolic pathways across species, their functional outcomes appear context-dependent. For instance, *Aspergillus fumigatus* CspA affects biofilm formation via a glycophosphatidylinositol-anchored cell wall protein [16], whereas *Xylella fastidiousa* Csp1 contributes to biofilm formation and long-term survival by influencing type IV pili subunit protein [17]. In contrast, *Listeria monocytogenes* Csp mutants display hyper-biofilm phenotypes [18], diverging from our observations.

Intriguingly, mycobacterial Csps universally attenuated oxidative stress resistance, as evidenced by enhanced hydrogen peroxide tolerance in Δ*cspA1*, Δ*cspA2*, and Δ*cspB* strains (Figure 3 and Figure 6). This contrasts with their protective roles in *E. coli* and *S. aureus*. For example, *E. coli* PprM, a cold shock domain-containing protein, activated antioxidant pathways via the *ycgZ*-*ymgABC* operon [19], independently of YcgF [20], *apo*-Fur [21], and TanA [22]. While the mechanistic divergence between PprM and mycobacterial Csps remains unresolved, our proteomic analysis of Δ*cspA1* revealed increased abundance of five oxidoreductases (e.g., tetrahydromethanopterin reductase, flavin-dependent oxidoreductases) and elevated WhiB3 expression (Figure S2, Table S2) [23]. This correlation suggests a potential link between CspA1 and oxidative homeostasis pathways, though direct regulatory relationships require further validation.

The absence of CspA1 correlated with altered INH sensitivity, potentially through *katG* expression changes. As INH requires activation by KatG to inhibit InhA (enoyl-ACP reductase) [24,25,26], this regulatory axis represents a potential contributor to mycobacterial drug resistance. While Csps influence antibiotic tolerance in other species through disparate pathways, e.g., *Salmonella Typhimurium* YciF, a CspE target, alters porin expression, and *Staphylococcus aureus cspB* mutant elevated superoxide dismutase levels to resist aminoglycosides, trimethoprim-sulfamethoxazole, and paraquat [27]. In addition, *Aspergillus fumigatus* CspA decreased drug resistance through influencing biofilm formation [16]. Our data highlight CspA1’s potential involvement in mycobacterial INH resistance through (1) modulation of KatG expression levels and (2) altered membrane permeability. Proteomic profiling indicated downregulated membrane proteins and upregulated MMPL family transporters in Δ*cspA1*, suggesting CspA1 may limit intracellular INH accumulation by impairing drug influx and activating efflux pumps.

## 5. Conclusions

In conclusion, our study establishes phenotypic associations between mycobacterial Csps and key persistence mechanisms, e.g., biofilm formation, oxidative stress tolerance, and INH resistance. We demonstrate that *CspA1* and *CspB* deletions correlate with impaired biofilm maturation but enhanced oxidative stress tolerance, a functional divergence from homologs in other bacteria. The absence of CspA1 further coincided with INH resistance through putative KatG-dependent and membrane-associated mechanisms. While our proteomic analyses revealed correlative metabolic pathways, future studies should determine whether Csps directly regulate these pathways or exert indirect effects through RNA chaperoning. These findings fill critical knowledge gaps regarding stress adaptation mechanisms in understudied mycobacterial Csps.

## Data Availability

The original contributions presented in this study are included in the article/Supplementary Materials. Further inquiries can be directed to the corresponding authors.

## Supplementary Materials

The following supporting information can be downloaded at: https://www.mdpi.com/article/10.3390/microorganisms13071597/s1, Figure S1: Proteomic analysis of Δ*cspA1* strain; Figure S2: The protein expression level of whiB3; Figure S3: The amino acid sequence of CspA1 is conserved in mycobacteria; Table S1: Primer details for Csps homologous arm amplification; Table S2: Upregulation levels of 5 oxidoreductases in the ΔcspA1 strain.
