# Supplementary material for: Cold Shock Proteins Balance Biofilm-Associated Antibiotic Resistance and Oxidative Vulnerability in Mycobacteria
Source: Microorganisms. 2025 Jul 7;13(7):1597. doi: 10.3390/microorganisms13071597 (PMC12300830; doi:10.3390/microorganisms13071597)
Supplement: Supplementary file 1 [file microorganisms-13-01597-s001.zip › microorganisms-3695834-supplementary.pdf]

Cold Shock Proteins Balance Biofilm-Associated Antibiotic Resistance and Oxidative Vulnerability in Mycobacteria

Jiachen Zheng, Linzhao He, Yizhang Wei, Jie Lu, Xiaolin Liu * and Weihui Li *

State Key Laboratory for Conservation and Utilization of Subtropical Agro-Bioresources, College of Life
Science and Technology, Guangxi University, Nanning 530004, China

* Correspondence: xiaolinliu@gxu.edu.cn (X.L.); lwhlbx@163.com (W.L.); Tel.: +86-771-2852965 (W.L.)

A list of the supplementary materials:

Figure S1. Proteomic analysis of Δ*cspA*1 strain.

Figure S2. The protein expression level of whiB3.

Figure S3. The amino acid sequence of CspA1 is conserved in mycobacteria.

Table S1. Primer details for Csps homologous arm amplification.

Table S2. Upregulation levels of 5 oxidoreductases in the Δ*cspA1* strain.

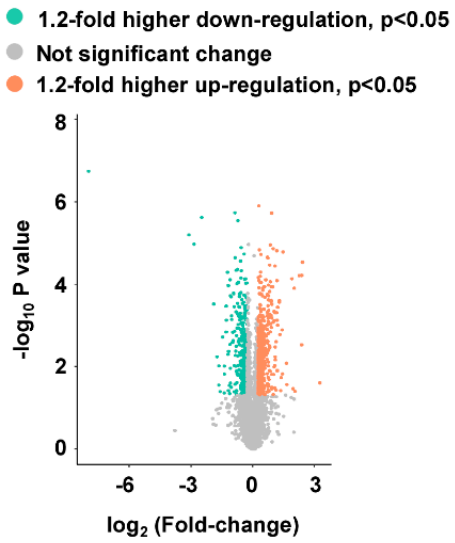

Figure S1. Proteomic analysis of Δ*cspA*1 strain. (A) Analysis of volcanic map of Δ*cspA*1 strain. Orange symbol: upregulation of expression by 1.2 times. Blue symbol: downregulation of expression by 1.2 times. The gray symbol: not significant change.

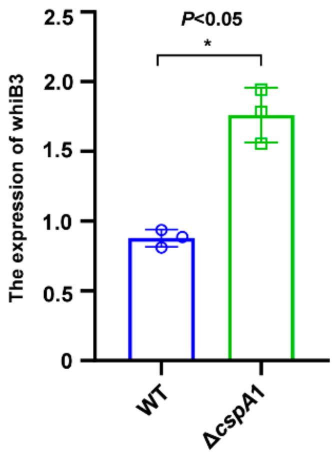

Figure S2. The protein expression level of whiB3. The protein expression levels of whiB3 in wild-type and Δ*CspA*1 strain. Error bars represent the variant range of the data derived from three biological replicates. Two-tailed Student's *t*-tests were performed for statistical analysis of three independent biological experiments (*P*<0.05, *).

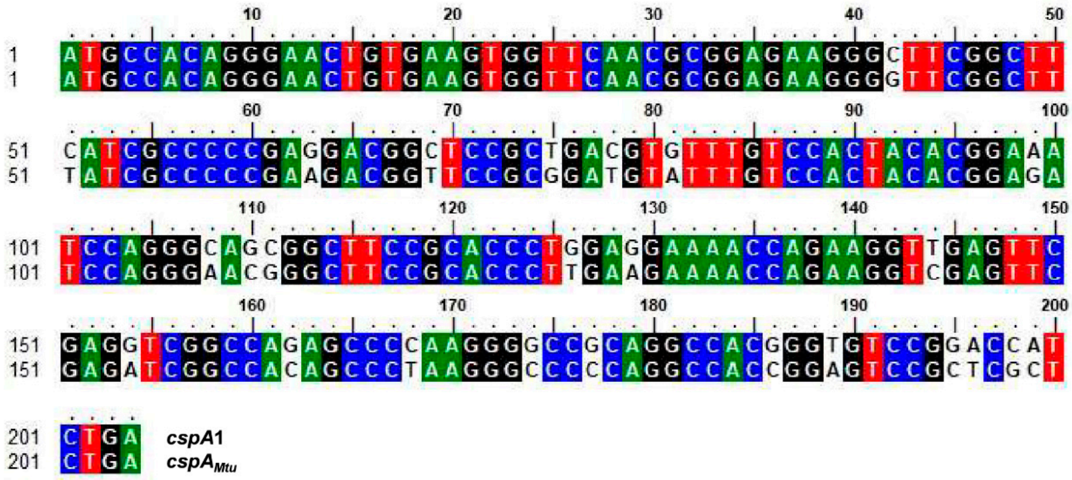

Figure S3. The amino acid sequence of CspA1 is conserved in mycobacteria. Amino acid sequence alignment of CspA1$_{Msm}$ and CspA1$_{Mtu}$. The same color in each column represents the same amino acid, and one color represents the type of amino acid.

Table S1. Primer details for CSPs homologous arm amplification.

Gene Name	Primer sequences
**pMV261-*MSMEG_6159* (*cspA1*) F**	TTATGAATTCATGCCACAGGGAACTGTGAAGTGG
**pMV261-*MSMEG_6159* (*cspA1*) R**	TAATTCTAGATCAGATGGTCCGGACACCCGTGGCC
**pMV261-*MSMEG_0559* (*cspA2*) F**	TTATGAATTCATGGCACAGGGAACTGTGAAATG
**pMV261-*MSMEG_0559* (*cspA2*) R**	TAATTCTAGATCAGACCGCGGTAACGCCAACTGCC
**pMV261-*MSMEG_5696* (*cspB*) F**	TTATGAATTCGTGTTTTTGTTGGCCGACAACGACG
**pMV261-*MSMEG_5696* (*cspB*) R**	TAATTCTAGATCAAGCGTCGAGTTCGCGCGCGACGGC
***cspA1* up F**	TAATTTAATTAATGGAGAAGATCCAGCGTGACCGTT
***cspA1* up R**	TAATACTAGTCACTTCTCGATATTTCCTTCTCTTCTT
***cspA1* down F**	TAAGAAGCTTGCGAGACAAACCTGACAGCA
***cspA1* down R**	TAATGCTAGCCCAGCCAACTGTGCCTACGCT
***cspA2* up F**	TAATTTAATTAATGTTCGGCTCGGGCATCAGCCCCGGCTA
***cspA2* up R**	TAATACTAGTCACATGTTTACTTCTCTCATTCAAT
***cspA2* down F**	TAAGAAGCTTTCGACCCCCAGACGTAACA
***cspA2* down R**	TAATGCTAGCGCGAAATCTTCTTCGCAC
***cspB* up F**	TAATTTAATTAAGATGCCGAGCCGGGCCACCGCGAT
***cspB* up R**	TAATACTAGTCACACAAAGTTCGCGTCCCGTCTGCGC
***cspAB* down F**	TAAGAAGCTTTCGTCCGAGTCCCGGGCGCGACCG
***cspB* down R**	TAATGCTAGCCTGAGCTTGGACCGGTTGCACT

Table S2. Upregulation levels of 5 oxidoreductases in the Δ*cspA1* strain.

Protein description	Gene name	CspA1/WT Ratio
Glucose-methanol-choline oxidoreductase	MSMEG_1428	1.538
Oxidoreductase (MocA family protein)	MSMEG_3300	1.642
Molybdopterin oxidoreductase	MSMEG_6816	1.702
Glucose-methanol-choline oxidoreductase	MSMEG_5967	2.565
Oxidoreductase (nitropropane dioxygenase family protein)	MSMEG_1660	2.497
